# The Book World of Medicine and Science

**Published:** 1902-01-25

**Authors:** 


### THE BOOK WORLD OF MEDICINE AND SCIENCE.

SMOKE AND ITS DIMINUTION. By BRYAN DONKIN, M.I.C.E., M.I.M.E. Reprinted from the *Engineer*. (Price 6d.)

THIS little pamphlet gives a good account of the manner in which smoke is produced, and of the various measures employed in preventing its occurrence. Whatever else he shows its author makes it evident that the problem presents considerable difficulties, and that although it is possible to make perhaps any furnace burn without producing smoke, this can only be done by the exercise of a considerable amount of care and skill, or by the use of apparatus which itself requires skill for its proper management. In any academic discussion on the subject it may be truly maintained that smoke can be consumed and that by consuming it the coal can in many cases be burned to greater advantage than when so much carbon is flying out of the top of the chimney; but it is not entirely true to say without reservation, as so many people do, that all this smoke production which we see going on is sheer waste. What a manufacturer wants out of his furnace is not merely to produce heat with the greatest possible economy, but to produce it when he requires it—which in many cases is not by any means equally all day long. In certain operations, such as the pumping of water, and in certain textile industries in which from morning till night the same force has to be exerted and exactly the same speed maintained, it may be easy enough, where the furnace and the boiler are of proper size, to avoid making smoke. It is done every day, and often requires nothing more than careful stoking; while in other trades, such as dyeing, where, without a moment's notice, great volumes of steam may be required for boiling up the vats, or in electric-light works where a slight wave of fog passing over the town may in a few moments double or treble the demand for light and therefore for steam, hard firing, and its consequence of smoke production, may be absolutely necessary unless a very large, and for all other purposes than smoke prevention, a uselessly large amount of boiler space and firegrate be provided. In such cases the choice often lies between wastefulness during most of the day, and smoke for an hour or two, and thus it may be true economy in some cases to make smoke. This should be understood. Manufacturers are not quite the fools that some people think them to be. If the making of smoke was the pure, sheer, unadulterated waste that some people make out, no compulsion on the factory owners would be necessary. They would be glad enough to economise. It is because they really save by making smoke that compulsion is required. But all this smoke production which the boiler-owners indulge in for their own advantage throws immense trouble and expense upon everyone else. Dirt, fog, want of light, and much bronchial disease are undoubtedly due to the smokiness of our atmosphere, and the community is fully in the right in insisting upon the abolition of this great nuisance. To come back now to our author, he advises various means for preventing the production of smoke, but he especially insists upon the necessity of a better system of inspection, urging that smoke inspectors should adopt a "smoke scale" such as is in use in Switzerland and record their observations on diagrams. At present, he says, "smoke inspectors often watch a certain chimney for about half an hour to one hour, and note down the different shades of smoke seen, according to their own judgment, without anything to aid them." Mr. Donkin, however, would, in important cases, have two observers placed at a considerable distance apart, and not in communication with each other, and let them plot on their diagrams the varying intensities of the smoke they observe each consecutive minute. Such "smoke diagrams" would be valuable evidence in case of a prosecution far more valuable than the vague evidence so often given on the subject under present conditions.

A POCKET CYCLOPEDIA OF MEDICINE AND SURGERY, BASED UPON "A CYCLOPEDIA OF PRACTICAL MEDICINE AND SURGERY." Edited by GEORGE M. GOULD, A.M., M.D., and WALTER L. PYLE, A.M., M.D. (Philadelphia: P. Blakiston's, Son and Co. 1902. Price one dollar.)

THE larger work on which this is based was an admirable boil down of the whole art and mystery of medicine and surgery, and this little volume is a still further and even more concentrated extract of these arts and sciences, and is equally admirable in its own way. What is the exact position which is to be taken by such a book, by whom it is to be read, and why anyone who has access to more complete sources of information should rush to pocket encyclopedias, we do not pretend to know. Granting, however, that the publishers have well gauged the public wants, there can be no doubt about the ability with which the editors have met the demand. A careful comparison with various larger works shows at once that a good deal of space has been gained by absolute omissions, while many of the subjects dealt with are only very slightly touched upon. For its size, however, it is very good, and so far as we have seen it contains but few errors. In the table giving the "differential diagnosis of the exanthemas," however, it is stated that the eruption of variola appears on the "fourth day," which is evidently wrong. The spelling is American, as in anemia, fetus, edema, gonorrhea, dysmenorrhea, diarrhea, which, perhaps, may not much matter. To all who want a pocket medical encyclopedia this handy little volume will prove very useful. It certainly is cram full of very well selected matter; no space is wasted, and every line is made to tell.

THE LONDON MANUAL FOR 1902. Edited by ROBERT DONALD. (London: Edward Lloyd, 1902. Price 1s. and 1s. 6d.)

As London grows larger, so does this manual year by year become a more substantial and more important volume. A very large amount of information is given in it on a multitude of subjects concerning London and its government; all is well brought up to date, and from the arrangement of its contents the book forms a very useful annotated and illustrated directory to official London. In this year's edition much attention is given to the various schemes which are afoot for improving the means of locomotion in the metropolis, and several maps are inserted showing the routes taken by the proposed railways, tubes, and tram-lines. Police, poor law, telephones, hospitals, theatres, magistrates, county council, and the numerous borough councils by which the local government of London is carried on, all come in for notice, and from the ample lists of officials which are given this volume has become almost essential to all who wish to take a part in the municipal life of the metropolis, as all good citizens thereof ought to do.

